# Decreased hormonal sensitivity after childbirth rather than the tumor size influences the prognosis of very young breast cancer patients

**DOI:** 10.1186/s40064-015-1150-0

**Published:** 2015-07-22

**Authors:** Masujiro Makita, Takehiko Sakai, Akemi Kataoka, Dai Kitagawa, Akiko Ogiya, Hidetomo Morizono, Yumi Miyagi, Kotaro Iijima, Kokoro Kobayashi, Takayuki Kobayashi, Ippei Fukada, Kazuhiro Araki, Shunji Takahashi, Yoshinori Ito, Naoya Gomi, Masahiko Oguchi, Mizuho Kita, Masami Arai, Futoshi Akiyama, Takuji Iwase

**Affiliations:** Department of Breast Surgical Oncology, Cancer Institute Hospital, 3-8-31 Ariake Koto-ku, Tokyo, 135-8550 Japan; Department of Breast Medical Oncology, Cancer Institute Hospital, Tokyo, Japan; Department of Diagnostic Radiology Center, Cancer Institute Hospital, Tokyo, Japan; Department of Radiation Oncology, Cancer Institute Hospital, Tokyo, Japan; Department of Clinical Genetic Oncology, Cancer Institute Hospital, Tokyo, Japan; Department of Pathology, Cancer Institute Hospital, Tokyo, Japan; Department of Breast Surgery, Nippon Medical School Musashi Kosugi Hospital, 1-396 Kosugi-machi, Nakahara-ku, Kawasaki, Kanagawa 211-8533 Japan

**Keywords:** Breast cancer, Younger age, Prognosis, Hormone receptor, Pregnancy-associated breast cancer

## Abstract

**Purpose:**

There is a significant difference in the mean tumor size between very young breast cancer patients and their elder counterparts. A simple comparison may show obvious prognostic differences. We investigated the prognostic impact of age by reducing the influence of the tumor size, which is thought to be a confounding factor.

**Patients and methods:**

We investigated 1,880 consecutive pT1-4N0-3M0 breast cancer patients treated at less than 45 years of age between 1986 and 2002 and conducted a case–control study of breast cancer subjects less than 30 years of age. Each patient (Younger than 30) was matched with a corresponding control subject (Elder counterpart) based on an age 15 years above the patient’s age, a similar tumor size and a status of being within 1 year after surgery. In addition, we assessed 47 patients with pregnancy-associated breast cancer (PABC). The levels of hormone receptors were measured using an enzyme immunoassay (EIA), and receptor-positive cases were divided into “weakly” and “strongly” positive groups based on the median value. Years from the last childbirth (YFLC) was categorized as “recent” and “past” at the time point of 8 years.

**Results:**

There were fewer past YFLC cases, more partial mastectomy cases, a higher rate of scirrhous carcinoma or solid-tubular carcinoma in the Younger than 30 group than in the Elder counterpart group. The rates of a PgR-negative status in the Younger than 30 and Elder counterpart groups were 45.1 and 29.9%, respectively, As for the relationship between the PgR-negative rate and YFLC, the rates of a PgR-negative status in the past YFLC, nulliparous, recent YFLC and PABC groups were 31.9, 37.7, 44.4 and 65.7%, respectively. On the other hand, the rates of strongly positive cases were 42.6, 30.2, 22.2 and 8.6%, respectively. The 10-year recurrence-free survival rates in the Younger than 30, Elder counterpart and PABC groups were 61.7, 65.6 and 54.1%, respectively. The differences between the groups were not significant. In a multivariate analysis, independent prognostic facers included the number of lymph node metastases (4–9, HR:3.388, 95% CI 1.363–8.425, p = 0.0086, over 10, HR: 6.714, 2.033–22.177, p = 0.0018), solid-tubular carcinoma (HR 3.348, 1.352–8.292, p = 0.0090), scirrhous carcinoma (HR 2.294, 1.013–5.197, p = 0.0465) and past YFLC (HR 0.422, 0.186–0.956, p = 0.0387). An age younger than 30 was not found to be an independent prognostic factor.

**Conclusions:**

The prognosis of the very young women was the same as their elder counterparts with a matched tumor size, and age was not identified to be an independent prognostic factor according to the multivariate analysis. Recent childbirth probably influences the prognosis of patients younger than 30 years of age with breast cancer by lowering hormonal sensitivity.

## Background

The poor prognosis of very young breast cancer patients has been reported to be caused partly by a delay in diagnosis (Maggard et al. 2003; Kataoka et al. 2014), and results are inconsistent as to whether age is an independent prognostic factor (Cancello et al. 2010; Crowe et al. 1994). On the other hand, some reports have found that the number of years from the last childbirth influences the prognosis of patients with breast cancer as well as pregnancy-associated breast cancer (PABC) (Mohle-Boetani et al. 1988; Kroman et al. 1997; Kroman and Mouridsen 2003; Nagatsuma et al. 2013). It is difficult to study the prognosis of very young breast cancer patients due to the paucity of patients and larger tumors. There is a significant difference in the mean tumor size between very young breast cancer patients and their elder counterparts. A simple comparison may show obvious prognostic differences. Therefore, it should be proven whether very young breast cancer patients have a poorer prognosis than their elder counterparts for tumors in the same stage.

Is the solution to improving the worse prognosis of very young breast cancer patients early detection only? Pregnancy experienced at a young age influences the prognosis of breast cancer, and the hormonal milieu of very young women differs from that observed in elder women, even those who are premenopausal. Some patients with a family history of breast cancer are apt to develop early-onset disease. It is therefore valuable to investigate important prognostic factors other than the tumor size when considering treatment for very young breast cancer patients. Hence, we investigated the prognostic impact of age by reducing the impact of the tumor size, which is thought to be a confounding factor.

## Patients and methods

A total of 9,713 consecutive patients were surgically treated for primary breast cancer between 1986 and 2002 at the Cancer Institute Hospital, Tokyo, Japan. The patients included in this study comprised only those who had been treated at less than 45 years of age at the time of surgery for breast cancer. Patients with distant metastases and noninvasive breast carcinoma, bilateral second breast cancer or synchronous bilateral breast cancer were excluded. In total, 1,880 individuals met the eligibility criteria for this study. We conducted a case–control study of very young (<30 years of age) breast cancer patients. Each very young breast cancer patient (younger than 30) was matched with a corresponding control patient (elder counterpart) in accordance with the following criteria: (1) an age 15 older than the patient’s age (e.g., if the patient was 23 y.o., the control was 38 y.o.), (2) a similar tumor size (pathological or clinical) and (3) a similar calendar year of breast surgery (within 1 year). We decided 15-years older cohort as Elder counterpart, because the difference of recurrence-free survival (RFS) between 15-years older cohort and the Younger than 30 was the largest, after comparing four RFS curves of 5-, 10-, 15-years older cohort and the Younger than 30. In addition, we assessed 47 patients with pregnancy-associated breast cancer (PABC: defined during pregnancy or within 1 year from childbirth) within the same study period. This study was approved by Institutional Review Board of Cancer Institute Hospital of Japanese Foundation for Cancer Research (2014-1115). The concentrations of hormone receptor were measured using an enzyme immunoassay (EIA). An estrogen receptor (ER)-positive status was defined as ≥5 fmol/mg and a progesterone receptor (PgR)-positive status was defined as ≥10 fmol/mg. Receptor-positive cases were divided into “weakly” and “strongly” positive groups based on the median value (the median ER and PgR values were 21 and 95, respectively). Years from the last childbirth (YFLC) was classified as “recent” or “past” at the time point of 8 years, because it was calculated as the cutoff point according to the receiver-operator curve (ROC) between the YFLC and recurrence/deaths groups using 119 parous cases. Because seven YFLC was the longest in the Younger than 30, it was meaningless to define 9 years and over as the cutoff point. And the correlation between recurrence/deaths and YFLC was not significant at the time point of 5 years, but significant at 8 years.

The following factors were evaluated: calendar year of surgery, family history of breast cancer, YFLC, tumor size (pathological), lymph node metastases, histological type classified according to the Japanese Breast Cancer Society: General Rules for Clinical and Pathological Recording of Breast Cancer guidelines (Japanese Breast Cancer Society 1989), extent of tumor invasion (Japanese Breast Cancer Society 1989), lymphovascular invasion, hormone receptor status, type of surgery and adjuvant treatment. We reviewed the patients’ charts retrospectively. All types of recurrence and death were considered as events, and RFS was calculated based on the Kaplan–Meier method. The onset of second breast cancer was considered to be censored in the heterochronous bilateral breast cancer cases. The univariate statistical analysis was performed using the Chi square test, Mann–Whitney U-test and log-rank test. The multivariate analysis was performed using Cox’s proportional hazard model. A p value of <0.05 was defined as significant. The computer software program, “Stat View for Windows version 4.54 “(Abacus Concepts, Inc., Berkley, CA, USA), was used for all analyses. The median follow-up time was 10.8 years.

## Results

The number of events in the Younger than 30 and Elder counterpart groups was 37 and 30, respectively. Except for one death without disease, all events were episodes of recurrence. The number of local and/or regional recurrence cases in the Younger than 30 and Elder counterpart groups was 15 and 13, respectively. The rates of distant metastases only per all recurrent cases were the same between the groups (younger than 30: 21/37 = 56.8%; elder counterpart: 17/30 = 56.7%). The number of heterochronous bilateral breast cancer cases was seven, all of which belonged to the younger than 30 group.

As for the case distribution, there were fewer parous cases, fewer past YFLC cases, more partial mastectomy cases, and higher rates of scirrhous carcinoma and solid-tubular carcinoma in the Younger than 30 group than in the Elder counterpart group (Table 1). The mean age in the Younger than 30, Elder counterpart and PABC groups was 27.1, 42.1 and 35.4 years, respectively. There were no differences between the two groups in terms of family history. Regarding hereditary breast and ovarian cancer (HBOC), there were no patients with *BRCA* mutations. Only two patients underwent genetic tests, and only one patient in the Younger than 30 group had a *p53* mutation and was diagnosed with Li-Fraumeni syndrome (Li and Fraumeni 1969).

**Table 1:** Patient characteristics

Factors	Case (younger than 30)	Control (elder counterpart)	Chi square test	PABC 47 cases
	Cases (%)	Cases (%)	P value	Cases (%)
Calendar year of surgery				
1986–1995	40 (50.0%)	40 (50.0%)	>0.9999	25 (53.2%)
1996–2002	40 (50.0%)	40 (50.0%)		22 (46.8%)
Age at surgery, years old				
<35	80 (100.0%)	1 (1.3%)	<0.0001	20 (42.6%)
≥35	0 (0.0%)	79 (98.8%)		27 (57.4%)
Family history of breast cancer				
None	69 (86.3%)	69 (86.3%)	>0.9999	40 (85.1%)
Positive	11 (13.8%)	11 (13.8%)		7 (14.9%)
Years from the last childbirth (YFLC)				
Nulliparous	68 (85.0%)	16 (20.0%)	<0.0001	4 (8.5%)
Recent (<8)	12 (15.0%)	8 (10.0%)		43 (91.5%)
Past (≥8)	0 (0.0%)	56 (70.0%)		0 (0.0%)
Tumor size, cm (Pathological)				
≤2	20 (25.0%)	23 (28.8%)	0.1826	13 (27.7%)
2.1–5	28 (35.0%)	42 (52.5%)		21 (44.7%)
>5	11 (13.8%)	6 (7.5%)		5 (10.6%)
The number of metastatic lymph nodes				
None	40 (50.0%)	38 (47.5%)	0.3553	14 (29.8%)
1–3	24 (30.0%)	23 (28.8%)		15 (31.9%)
4–9	6 (7.5%)	13 (16.3%)		11 (23.4%)
10–	9 (11.3%)	6 (7.5%)		7 (14.9%)
Histological type				
Papillotubular carcinoma	18 (22.5%)	24 (30.0%)	0.1003	9 (19.1%)
Sollid-tubular carcinoma	28 (35.0%)	16 (20.0%)		20 (42.6%)
Scirrhous carcinoma	29 (36.3%)	27 (33.8%)		13 (27.7%)
Special types	4 (5.0%)	10 (12.5%)		5 (10.6%)
Unilateral double cancer	1 (1.3%)	3 (3.8%)		0 (0.0%)
Extent of tumor invasion (histological)				
Localized within mammary gland	29 (36.3%)	23 (28.8%)	0.4486	22 (46.8%)
Invading the extramammary fat tissue	47 (58.8%)	50 (62.5%)		21 (44.7%)
Invading the skin and/or muscle	4 (5.0%)	7 (8.8%)		4 (8.5%)
Lymphovascular invasion				
Absent	45 (56.3%)	53(66.3%)	0.2559	24 (51.1%)
Present	35 (43.8%)	27(33.8%)		23 (48.9%)
Estrogen receptor(EIA), fmol/mg				
<5 (negative)	31 (38.8%)	30 (37.5%)	0.2563	31 (66.0%)
5–21 (weakly positive)	14 (17.5%)	18 (22.5%)		2 (4.3%)
22– (strongly positive)	9 (11.3%)	19 (23.8%)		4 (8.5%)
Not performed	26 (32.5%)	13 (16.3%)		10 (21.3%)
Progesterone receptor(EIA), fmol/mg				
<10 (negative)	23 (28.8%)	20 (25.0%)	0.1555	23 (48.9%)
10–95 (Weakly positive)	15 (18.8%)	20 (25.0%)		9 (19.1%)
96– (Strongly positive)	13 (16.3%)	27 (33.8%)		3 (6.4%)
Not performed	29 (36.3%)	13 (16.3%)		12 (25.5%)
Type of breast surgery				
Breast conserving surgery (BCS)	27 (33.8%)	16 (20.0%)	0.0738	6 (12.8%)
Mastectomy	53 (66.3%)	64 (80.0%)		41 (87.2%)
Chemotherapy				
None	24 (30.0%)	28 (35.0%)	0.3417	12 (25.5%)
Others	5 (6.3%)	10 (12.5%)		8 (17.0%)
CMF	33 (41.3%)	31 (38.8%)		14 (29.8%)
Anthracycline	13 (16.3%)	8 (10.0%)		8 (17.0%)
Anthracycline and Taxane	5 (6.3%)	2 (2.5%)		5 (10.6%)
Hormone therapy				
Ovarian function suppression ± others	11 (13.8%)	7 (8.8%)	0.0007	4 (8.5%)
Selective estrogen receptor modulators	8 (10.0%)	28 (35.0%)		10 (21.3%)
Others, none	61 (76.3%)	45 (56.3%)		33 (70.2%)
Radiation therapy (RT)				
Not performed	63 (78.8%)	68 (85.0%)	0.3149	42 (89.4%)
Performed	17 (21.3%)	12 (15.0%)		5 (10.6%)

**Figure 1:**
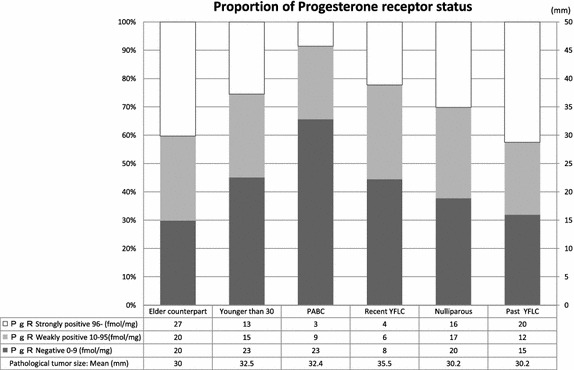
Progesterone receptor status in each group. Age or more recent childbirth was correlated with lower hormonal sensitivity. (*YFLC* years from the last childbirth).

**Figure 2:**
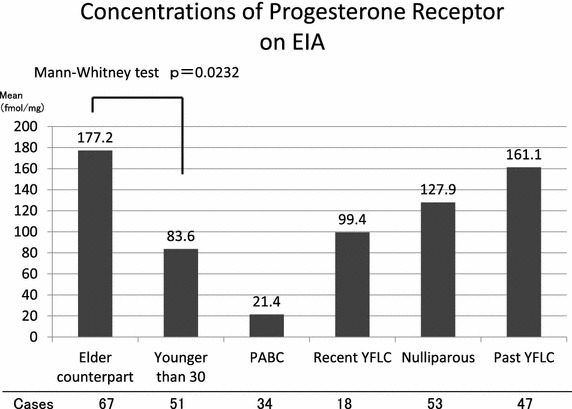
Concentrations of progesterone receptor on EIA. The concentrations of PgR on EIA were significantly lower in the younger than 30 group than in the elder counterpart group (Mann–Whitney U test p = 0.0232).

The rates of an ER-negative status in the Younger than 30 and Elder counterpart groups were 57.4% (31/54) and 44.8% (30/67), respectively, while the rates of a PgR-negative status in the Younger than 30 and Elder counterpart groups were 45.1% (23/51) and 29.9% (20/67), respectively. As for the relationship between the PgR-negative rate and YFLC, the rates of a PgR-negative status in the past YFLC, nulliparous, recent YFLC and PABC groups were 31.9, 37.7, 44.4 and 65.7%, respectively. On the other hand, the rates of strongly positive findings (>95 fmol/mg) were 42.6, 30.2, 22.2 and 8.6%, respectively (Figure 1). More recent childbirth was correlated with lower hormonal sensitivity. The mean ER values on EIA in the Younger than 30, Elder counterpart and PABC groups were 10.9, 18.9 and 4.2 fmol/mg, respectively and the mean PgR values in the Younger than 30, Elder counterpart and PABC groups were 83.6, 177.2 and 21.4 fmol/mg, respectively; the concentrations of PgR on EIA were significantly lower in the Younger than 30 group than in the Elder counterpart group (Mann–Whitney U test p = 0.0232, Figure 2).

**Figure 3:**
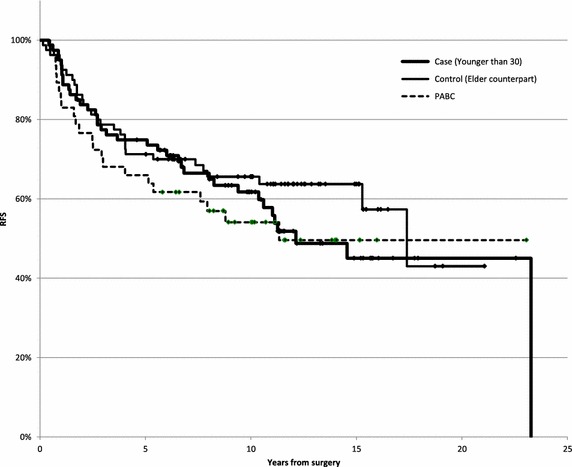
RFS curves in the younger than 30, Elder counterpart and PABC groups. Although the RFS curve in the younger than 30 group gradually decreased after 10 years, the difference between the groups was not significant.

The RFS rate was 61.7% at 10 years and 45% at 15 years in the Younger than 30 group. On the other hand, these rates in the Elderly counterpart group were 65.6 and 63.7%, respectively (p = 0.3865, Log-rank test, Figure 3) and those in the PABC group were 54.1 and 49.6%, respectively. Although the RFS curve in the younger than 30 group gradually decreased after 10 years, the difference between the groups was not significant.

The results of the univariate analysis of each factor among the total 160 cases in the younger than 30 and Elderly counterpart groups are demonstrated in Table 2. The 10-year RFS in the past YFLC group was 72%, which was significantly higher than that seen in the nulliparous/recent YFLC group (59.3%, p = 0.0399). The multivariate analysis of significant factors identified in the univariate analyses (tumor size, lymph node metastases, histological type, extent of tumor invasion, lymphovascular invasion, adjuvant chemotherapy and PgR), in addition to age and YFLC, showed the independent prognostic facers to be the number of lymph node metastases (4–9, HR:3.388, 95% CI 1.363–8.425, p = 0.0086, over 10, HR: 6.714, 2.033–22.177, p = 0.0018), solid-tubular carcinoma (HR 3.348, 1.352–8.292, p = 0.0090), scirrhous carcinoma (HR 2.294, 1.013–5.197, p = 0.0465) and past YFLC (HR 0.422, 0.186–0.956, p = 0.0387. Table 2). An age younger than 30 years was not found to be an independent prognostic factor.

**Table 2:** Univariate and multivariate analyses

Factors	Univariate analysis				Cox’s proportional hasard model			
				Logrank test		95% CI		
	Cases	Recurrence/died	10 year RFS (%)	P value	HR	Lower limit	Upper limit	P value
Calendar year of surgery								
1986–1995	80	35	65.7	0.5963				
1996–2002	80	32	61.0					
Family history of breast cancer								
None	138	58	64.9	0.9870				
Positive	22	9	56.9					
Years from the last delivery (YFLC)								
Nulliparous	84	42	59.4	0.1208	1			
Recent (<8)	20	9	58.7		0.731	0.317	1.686	0.4618
Past (≥8)	56	16	72.0		0.422	0.186	0.956	0.0387
Past	56	16	72.0	0.0399				
Nulliparous/Recent	104	51	59.3					
Tumor size, cm (Pathological)								
≤5	113	43	67.0	<0.0001	1			
>5	17	13	23.5		1.426	0.520	3.912	0.4911
The number of metastatic lymph nodes								
None	78	25	74.9	<0.0001	1			
1–3	47	17	72.1		1.812	0.805	4.079	0.1512
4–9	19	12	36.8		3.388	1.363	8.425	0.0086
10–	15	13	13.3		6.714	2.033	22.177	0.0018
Histological type^a^								
Papillotubular carcinoma	45	10	83.7	0.0149	1			
Sollid-tubular carcinoma	44	19	58.2		3.348	1.352	8.292	0.0090
Scirrhous carcinoma	57	30	55.4		2.294	1.013	5.197	0.0465
Special types	14	8	50.0		2.816	0.887	8.944	0.0791
Extent of tumor invasion (histological)								
Localized within gland or fat	149	60	65.7	0.0072	1			
Invading the skin and/or muscle	11	7	36.4		2.455	0.888	6.787	0.0834
Lymphovascular invasion								
Absent	98	33	71.7	0.0015	1			
Present	62	34	50.6		1.883	0.966	3.696	0.0629
Estrogen receptor(EIA), fmol/mg								
<5 (negative)	61	28	62.9	0.3093				
5–21 (weakly positive)	32	12	61.1					
22– (strongly positive)	28	13	53.1					
Not performed	39	14	76.0					
Progesterone receptor(EIA), fmol/mg								
<10 (negative)	43	21	56.7	0.1221				
10–95 (Weakly positive)	35	17	54.0					
96– (strongly positive)	40	14	68.8					
Not performed	42	15	75.0					
Negative/weakly positive	78	38	55.2	0.0189	1			
Strongly positive/Not performed	82	29	71.9		0.679	0.361	1.274	0.2278
Type of breast surgery								
Breast conserving surgery (BCS)	43	15	71.3	0.4737				
Mastectomy	117	52	61.3					
Chemotherapy								
Others, none	68	23	72.9		1			
Anthracycline/Taxane/CMF	92	44	56.8	0.0136	0.714	0.342	1.489	0.3689
Hormone therapy								
Ovarian Function Suppression ± Others	18	5	67.3	0.6537				
Selective estrogen receptor modulators	36	15	59.5					
Others, none	106	47	64.2					
Radiation therapy (RT)								
Not performed	131	54	65.6	0.2767				
Performed	29	13	53.7					
Age at surgery								
Case (younger than 30)	80	37	61.7	0.3865	0.557	0.251	1.239	0.1515
Control (elder counterpart)	80	30	65.6		1			

^a^Category “Unilateral double cancer” was re-classified to the histological type of the larger invasive tumor.

## Discussion

The frequency of breast cancers at less than 30 years of age is approximately 1% of all breast cancers, and a younger age has been reported to have a worse prognosis among premenopausal as well as all breast cancer patients (Maggard et al. 2003; Cancello et al. 2010). However, the recent recommendation of St. Gallen excluded a younger age as prognostic factor (Glick et al. 1992; Goldhirsch et al. 2001, 2009; Colleoni et al. 2006), and we previously reported that the prognosis of PABC is correlated with a younger age (Makita et al. 2007). In this study, a younger age was not found to be an independent prognostic factor, although recent childbirth probably influenced the prognosis of the younger than 30 breast cancer patients by lowering their hormonal sensitivity. In addition, the prognosis of PABC and very young breast cancer patients can be explained by considering the interval from the last childbirth (Johansson et al. 2013).

Factors such as Human Epidermal Growth Factor Receptor2 (HER2), Ki67 and the nuclear grade were not investigated in this study because these parameters were not routinely evaluated in the period of this case series. However, recurrence occurs earlier in hormone receptor-negative cases (HER2 subtype and triple negative breast cancer) than in cases of the luminal subtype (Metzger-Filho et al. 2013), and the timing of recurrence is strongly influenced by hormonal sensitivity (Makita et al. 2014). We believe that the trend in the recurrence-free interval can be explained by hormonal sensitivity alone, instead of based on the subtype. Even if additional data were available, the results would not change regarding the chief influencing factor being hormone sensitivity.

Although hormonal sensitivity is routinely evaluated based on the immunohistochemical method, we intended to investigate the EIA data exclusively due to the assessment to evaluate hormonal sensitivity quantitatively and objectively. The rates of a strongly positive PgR status (≥96 fmol/mg) differed based on YFLC. On the other hand, the analysis of old case series and longer follow-up period showed that the RFS curve in the Younger than 30 group gradually decreased, even after 10 years (Figure 3), whereas that in the Elder counterpart group reached a plateau. As for the relationship between the results for PgR and the prognosis, the RFS rate in the cases in which PgR EIA was not performed was as high as that noted in the cases with a strongly positive PgR status. These cases likely belong to an earlier stage, as the lesions were difficult to diagnose, except when performing an open biopsy, or were too small to obtain an adequate sample for EIA. PgR has been reported to be an important prognostic factor among cases of hormone sensitive breast cancer (Prat et al. 2013), and the PgR status was found to be related to the prognosis, rather than the ER status, in this study.

Whereas the number of years from the last birth was set at 8 years as the cutoff point calculated according to the ROC in this study, the prognosis of the cases within 2 years from the last childbirth has been reported to be worse (Mohle-Boetani et al. 1988; Kroman et al. 1997; Kroman and Mouridsen 2003; Nagatsuma et al. 2013). Despite the different cutoff points between previous and the present study, due to the limited number of cases in this study, the trends displayed in these studies were the same, and recent childbirth is thought to influence the prognosis of breast cancer. This finding is related to a report showing that childbirth conveys a long-term reduction in the incidence of breast cancer despite a transient, short-term increase in the incidence of such cancer (Lambe et al. 1994). Elevated levels of estrogens during pregnancy have been suggested to act as a promoter of premalignant breast cells, thus explaining the transient increase in risk after childbirth. It is probable that elevated levels of estrogens act as a stimulator of malignant breast cells, which explains the transient increase in recurrent risk after childbirth. Indeed, our data indicate that the prognostic influence of parity differs between the patients less than 35 years of age and the patients 35–44 years of age. Although the 10-year RFS rate of parous women was 49.0%, that of nulliparous women was 62.9% (p = 0.0152) among the cases less than 35 years of age. On the other hand, in the cases 35–44 years of age, the 10-year RFS rates of parous and nulliparous women were 75.4 and 77.6%, respectively. In younger women, parity has an adverse effect on the prognosis and this finding is related to a higher frequency of recent childbirth in younger women. Because childbirth conveys a long-term reduction in the rate of recurrence of breast cancer despite a transient, short-term increase in the frequency of recurrence, the time of 8 years from the last childbirth is considered to be the cutoff point for favorable effects rather than adverse effects.

The results showing that the case distribution of tumor size and number of lymph node metastases did not differ between the groups demonstrated that the effects of confounding factors between the two groups were successfully eliminated. Factors displaying differences between the groups included the histological type, type of breast surgery and YFLC. The rate of cases classified as solid-tubular carcinoma was higher in the Younger than 30 group than in the Elder counterpart group, and the histological type was found to be an independent prognostic factor as well as the number of lymph node metastases in the multivariate analysis. Solid-tubular carcinoma and scirrhous carcinoma are classified as poorly differentiated with a higher nuclear grade (Japanese Breast Cancer Society 1989) and have been reported to have a poor prognosis (Sakamoto 1989). It is thought that young patients (<35 years of age) with breast cancer are apt to develop poorly differentiated lesions and display more aggressive features (Maggard et al. 2003; Kataoka et al. 2014). On the other hand, the tumor size was not found to be an independent prognostic factor in the current study. The worse prognosis of very young breast cancer patients is thought to be related to tumor biology, including the histological features and hormone sensitivity.

The number of the cases treated with breast conserving surgery was larger in the Younger than 30 group than in the Elder counterpart group; however, the type of breast surgery was not identified to be an independent prognostic factor in this study. Although age has been reported to be an independent prognostic factor for ipsilateral breast tumor recurrence (Arvold et al. 2011), the number of cases of locoregional failure after surgery was almost the same in the two groups in the current study. Therefore, the difference in the type of surgery between the cases and controls did not necessarily influence the prognosis.

In the univariate analysis of the subgroup with 1–3 lymph node metastases, the rates of 10-year RFS in the Younger than 30 and Elder counterpart groups were 59.7 and 85.6%, respectively, and this difference was significant (p = 0.0329). As for the case distribution in this subgroup (24 cases in the Younger than 30 group, 23 cases in the Elder counterpart group), the rate of papillotubular carcinoma was lower and the number of cases in which a hormone receptor analysis was not performed was larger in the Younger than 30 group. The reason for not performing a hormone receptor analysis was related to the use of open biopsies before the diagnosis, mainly because the lesions were difficult to diagnose correctly in the Younger than 30 group. Due to the lack of hormone receptor status, these patients in the Younger than 30 group were treated with chemotherapy only and were thus treated insufficiently, although we had no data about chemotherapy-induced amenorrhea in these cases. It is probable that the decreased RFS in the younger women was caused by the use of inadequate hormone therapy, as mentioned in other reports (Colleoni et al. 2006). Another probable cause is that most of these cases were treated before approval was given for the use of luteinizing hormone releasing hormone agonist (LHRHA) as adjuvant therapy.

As for family history and HBOC, there were no patients with *BRCA* mutations, and only one patient in the Younger than 30 group had a p53 mutation and was diagnosed with Li-Fraumeni syndrome (Li and Fraumeni 1969) among the two patients who received genetic tests. Five patients had more than one case of breast cancer in their family in the Younger than 30 group; however, no cases were observed in the Elder counterpart group. Over 15 years, the patients in the Younger than 30 group had more cases of breast cancer or ovarian cancer in their families, and it is probable that HBOC cases were included in this group. However, such cases did not occupy the majority. Although the *BRCA1* mutation is related to triple negative breast cancer (Lee et al. 2011), a family history of breast cancer did not influence hormonal sensitivity in this study, as three of five cases in the Younger than 30 group were PgR-positive.

A correlation between recent childbirth and the prognosis of malignant disease has been reported and possible biological mechanisms of the adverse prognostic effect include immunosuppression, the hormonal milieu in gestation and a tumor promoting microenvironment post-partum (Moller et al. 2013). However, decreased hormone sensitivity after childbirth is a probable chief cause of adverse prognostic effects. Although collected data about the number of years from recent childbirth from 207 cases was limited in this study, the breast cancer patients who had given birth more recently showed an increased rate of PgR-negative tumors in another report (Nagatsuma et al. 2013). The correlation between recent childbirth and decreased hormone sensitivity was demonstrated from the point of intensity evaluated using EIA in this study. The prognosis of the very young women was the same as that for their elder counterparts, whose tumor size was matched, and age was not an independent prognostic factor according to the multivariate analysis. In conclusion, recent childbirth rather than the tumor size probably influences the prognosis of breast cancer patients younger than 30 years of age by lowering hormonal sensitivity.
